# KDM3B suppresses APL progression by restricting chromatin accessibility and facilitating the ATRA-mediated degradation of PML/RARα

**DOI:** 10.1186/s12935-019-0979-7

**Published:** 2019-10-04

**Authors:** Xinrui Wang, Huiyong Fan, Congling Xu, Guojuan Jiang, Haiwei Wang, Ji Zhang

**Affiliations:** 1 State Key Laboratory of Medical Genomics, Shanghai Institute of Hematology, Rui-Jin Hospital, Shanghai Jiao Tong University School of Medicine, Shanghai, 200025 China; 2 Institute of Health Sciences, Shanghai Institutes for Biological Sciences and Graduate School, Chinese Academy of Sciences, Shanghai, 200025 China

**Keywords:** KDM3B, APL, PML/RARα, H3K9me1/me2, Chromatin accessibility, Differentiation

## Abstract

**Background:**

A hallmark of acute promyelocytic leukemia (APL) is the expression of PML/RARα fusion protein. Treatment with all-trans retinoic acid (ATRA) results in the terminal differentiation of neutrophil granulocytes. However, the underlying mechanisms remain largely unknown. Here, we identify and elucidate a novel differentiation-suppressive model of APL involving the histone demethylase KDM3B, which has been identified as a suppressor of the tumor genes involved in hematopoietic malignancies.

**Methods:**

First, we established a KDM3B knockdown NB4 cell model to determine the functional characteristics of KDM3B by cell proliferation assay and flow cytometry. Then, we performed ChIP-seq and ATAC-seq to search for potential relationships among KDM3B, histone modification (H3K9me1/me2) and the chromatin state. Finally, molecular biological techniques and a multi-omics analysis were used to explore the role of KDM3B in differentiation of the leukemia cells after ATRA treatment.

**Results:**

We found that knocking down KDM3B contributed to the growth of NB4 APL cells via the promotion of cell-cycle progression and blocked granulocytic differentiation. Through global and molecular approaches, we provided futher evidence that knocking down KDM3B altered the global distribution of H3K9me1/me2 and increased the chromatin accessibility. Moreover, knocking down KDM3B inhibited the ATRA-induced degradation of the PML/RARα oncoprotein.

**Conclusion:**

Our study suggested that KDM3B was able to inhibit APL progression by maintaining chromatin in a compact state and facilitating the ATRA-mediated degradation of PML/RARα. Taken together, the results show that KDM3B may be an alternative target for the treatment regimens and the targeted therapy for APL by sustaining the function of PML/RARα fusion protein.

## Background

The epigenetic information encoded in chromatin is crucial for cell fate and differentiation [1–3]. Post-translational modifications in the histone tail are important epigenetic marks linked to transcriptional activation or repression of their specific target genes [4, 5]. One major type of chromatin-level regulation is histone methylation. In particular, histone lysine residues can be modified by mono-methylation (me1), dimethylation (me2), or tri-methylation (me3) [6]. Histone lysine methylation is linked to chromatin remodeling and its activity depends on the specific lysine targeted [7, 8]. The methylation of histone H3 lysine 9 (H3K9) is one of the most intensively studied histone modifications because it is considered an epigenetic marker of transcriptionally silenced heterochromatin [4, 9, 10].

Histone lysine methylation has been proven to be reversible and many histone methylation modified enzymes have been identified [4, 11]. In the developmental process, the status of H3K9 methylation is tightly controlled by the dynamic alternation of lysine methyltransferases and demethylases [12–14]. Among these enzymes, KDM3 family members contain a Jumonji C (JmjC) domain and possess intrinsic H3K9 demethylating activity [15]. The KDM3 family has three important members, namely, KDM3A (also known as JMJD1A, JHDM2A or TSGA), KDM3B (also known as JMJD1B, JHDM2B or 5qNCA) and JMJD1C (also known as JHDM2C or TRIP8) [16–18].

KDM3B is a H3K9me1/me2-specific demethylase that was initially suspected of inhibiting the tumor activity of hematopoietic malignancies due to its location in the 5q31 chromosome region, which is a frequently deleted region in myelodysplastic syndromes (MDS) and acute myeloid leukemia (AML) [16, 19–21]. Although, some results have suggested that KDM3B mediates H3K9 methylation, which plays important roles in AML development, progression and prognosis, due to the lack of global information on KDM3B and H3K9 methylation status of chromatin, the mechanisms of H3K9 methylation mediated by KDM3B in hematopoietic malignance development are not known [22–24].

Previously, our lab focused on the differentiation process in ATRA-mediated acute promyelocytic leukemia (APL) and identified a critical blocked differentiation mechanism in APL [25, 26]. Our results suggested that oncogenic fusion protein PML/RARα is bound to and represses the activation of the PU.1-mediated gene signature which is essential for myeloid differentiation. Taking advantage of our previous extensive studies on APL, we chose to study the epigenetic alterations that are induced by KDM3B-mediated H3K9 methylation during APL pathogenesis.

Thus far, the epigenetic mechanisms of H3K9 methyltransferases or demethylases have been implicated in the pathogenesis of hematopoietic malignancies, as determined with the information acquired through next-generation sequencing (NGS) [8, 27]. Namely, to thoroughly analyze, for the first time, the regulatory mechanisms of KDM3B that are involved in the ATRA-induced differentiation of NB4 cells, we performed a ChIP assay using antibodies that recognize H3K9me1/me2 and an ATAC assay. In the present study, we demonstrate that knocking down KDM3B leads to an increase in H3K9me1 levels and enhanced chromatin accessibility in transcription start site (TSS) regions of the global genome, which includes the PML/RARα target genes, whereas the levels of modified H3K9me2 are reduced in these regions. With the use of a series of experimental assays and using leukemia cell lines (NB4 and PR9), we proved that KDM3B inhibits the survival of APL NB4 cells and it is required for the differentiation of leukemia cells after ATRA treatment.

## Materials and methods

### Cell culture

PR9 and NB4 cells were grown in RPMI-1640 medium (GibcoBRL, Gaithersburg, MD, USA), supplemented with 10% FBS (GibcoBRL, Gaithersburg, MD, USA). Human 293T cells were grown in DMEM (GibcoBRL, Gaithersburg, MD, USA) supplemented with 10% fetal bovine serum. All cells were maintained at 37 °C in a humidified atmosphere containing 5% CO_2_. For induction of granulocytic differentiation, cells were incubated with 1 mM ATRA (Sigma, St. Louis, MO, USA). For induction of PML/RARa expression, PR9 cells were incubated with 100 mM ZnSO_4_ (Sigma, St. Louis, MO, USA) for 4 h.

### shRNA-induced gene silencing by lentiviral infection

Lentiviral-based vectors for RNA interference-mediated gene silencing were pLVX-shRNA2-Puro, containing shRNAs scrambled (against the sequence 5ʹ-GCCACAACGTCTATATCATGG-3ʹ), of KDM3B (against the sequence 5ʹ-GCGATCTTTGTAGAATTTGAT-3ʹ and 5ʹ-GCACTGAGAGAAACAGTTAAT-3ʹ). Lentiviral particles were produced in HEK293T (human embryonic kidney) cells for 72 h upon transfection of the shRNA vectors together with psPAX2 and pMD2G plasmids using polyenthyleneimine (Sigma). Medium was then centrifuged (1000×*g*, 5 min) and filtered through a 0.45 mm membrane (Millipore). Cells were incubated with medium containing lentiviral particles for 24 h. Then, medium was replaced to allow the knockdown for 2 or more days. pLVX-shRNA2-Puro vectors contain a puromycin resistance site. Puromycin (4 mg/mL; Sigma) was added to the media to select for resistant cells and to generate stable target-deficient cell lines and Polybrene (1.75 mg/mL; Sigma) to enhance viral infection.

### RNA isolation and quantitative PCR (qPCR)

Total RNA was isolated from the collected cells using RNeasy Mini-kit (Qiagen, Hilden, Germany) and reverse transcribed into cDNA that prepared by PrimeScript™ RT reagent Kit (TaKaRa, Tokyo, Japan) as the manufacturer’s directions. Relative RNA expression was normalized to β-actin using the 2^−ΔΔCt^ method [28]. QPCR was conducted as follows: 95 °C for 10 min; and 40 cycles of 95 °C for 15 s and 60 °C for 1 min. QPCR were performed on the ABI ViiA™ 7 Real-Time PCR system (Applied Biosystems) using SYBE‑Green qPCR Supermix (TOYOBO, Tokyo, Japan). Gene primer sequences were shown in Additional file 1.

### Protein extraction and immunoblots

Nuclear protein was extracted by using the NE-PER Nuclear and Cytoplasmic Extraction Reagents using the manufacturer’s instructions. For total protein extraction, the cells were collected and lysed in Lysis buffer (20 mM Tris/HCl pH 7.5; 120 mM NaCl; 1 mM EDTA; 1 mM EGTA; 1% Triton X-100; 2.5 mM Sodium pyrophosphate; 1 mM β Glycerophosphate; 1 mM Na_3_VO_4_) in the presence of protease inhibitors (Sigma-Aldrich). Individual samples were denatured and loaded onto 8–12% Tris Acrylamide gels and electrotransferred onto PVDF membranes (Millipore). After blocking with 5% skimmed milk in TBST for 2 h at room temperature, membranes were incubated at 4 °C overnight with specific primary antibodies with details shown in the supplement table. Subsequent to washing, membranes were incubated with horseradish peroxidase-conjugated (HRP) secondary antibodies for 2 h. Signals were developed by ECL reagent (Millipore) and captured by FluorChem M (ProteinSimple). Histone 3 and β-actin were used as markers of the nucleus and whole cell lysate, respectively. Details of antibodies were shown in the Additional file 1.

### Immunofluorescence

Cells were fixed in 4% paraformaldehyde, then washed with phosphate-buffered saline (PBS). Dispersed cells were attached to positively-charged slides using a CytoSpin and incubated in blocking solution (PBS with 0.3% Triton and 10% fetal bovine serum FBS) [19]. Primary antibodies were incubated overnight at 4 °C, and secondary antibodies were incubated at room temperature for 3 h. DAPI (4′,6-diamidino-2-phenylindole) was added with the mounting media to counterstain DNA. Pictures of random fields were taken and then analyzed on a Leica TCS SP8 confocal microscope. Details of antibodies were shown in the supplement Additional file 1.

### ChIP-Seq and bioinformatics analysis

ChIP assay was performed using the Active Motif ChIP-IT kit (Carlsbad, CA, USA) according to manufacturer’s instructions. Briefly, cells were lysed and sonicated after cross-linking by 1% formaldehyde for 10 min at room temperature. The crosslinked complex was immuno-precipitated by target antibody or control rabbit IgG (Cell Signaling Technology, Beverly, MA, USA) bound to protein A/G agarose beads. After overnight incubation at 4 °C, the complex was eluted and DNA was purified. ChIP products were submitted to Shanghai Personal Biotechnology. Libraries were created from successful large-scaled ChIP experiments using Illumina’s TruSeq Library Prep Kit following instructions in the user manual (Illumina, San Diego, CA, USA). The library size was estimated using a Bioanalyzer 2100. Pooled libraries were submitted for sequencing on the Illumina HiSeq X ten system at 150 bp pair-ended sequencing. ChIP-Seq reads were quality controlled and trimmed for adapter sequences using Trim Galore. Filtered reads were aligned to hg38 using Bowtie2. Sam files from Bowtie were converted into bam files by SAMtools. The profile and correlation plots were made with DeepTools. Visualization of read count data was performed by converting raw bam files to bigwig files using IGV tools. 3 kb up- and downstream regions of the TSS were used for TSS-based profile plots and 3 kb up- and downstream regions PML-RARα peaks were used for PML-RARα-based profile plots.

### ATAC-Seq and bioinformatics analysis

ATAC-seq was performed as described on harvested cell samples [29, 30]. Libraries were purified with AMPure beads (Agencourt) to remove contaminating primer dimers. The library size was estimated using a Bioanalyzer 2100. Pooled libraries were submitted for sequencing on the Illumina HiSeq X ten system at 150 bp pair-ended sequencing. All reads for each sample were combined and aligned to hg38 with bowtie2 [18]. Peaks were called for each sample using HOMER and individual peaks separated by < 500 bp were joined together. The peak annotation was assessed using the ‘ChIPseeker’ library from R/Bioconductor. Motif analysis on peak regions was performed by HOMER function. Heatmaps and profile plots were generated using DeepTools. Visualization of genome-wide read coverage was performed by converting raw bam files to bigwig files using IGV tools. 0.5 kb up- and downstream regions of the TSS were used for TSS-based heatmaps. 0.5 kb up- and downstream regions PML-RARα peaks were used for PML-RARα-based heatmaps.

### RNA-Seq and bioinformatics analysis

Total RNA prepared was extracted using TRIzol Reagent (Invitrogen, Carlsbad, CA, USA) and submitted to Shanghai Personal Biotechnology, where RNA intergrity was confirmed using the Illumina HiSeq X ten system at 150 bp pair-ended. Double-strand cDNA libraries were prepared and constructed using the TruSeq RNA Sample Prep Kit (Illumina, San Diego, CA). Two replicates of the RNA-Seq experiments were performed. RNA-Seq reads were quality controlled and trimmed for adapter sequences using Trim Galore. Filtered reads were aligned to hg38 using HISAT2. Read counts for each gene were carried out using HT-Seq using the hg38 refSeq refFlat GTF file accessed on July 2015. Differentially expressed genes (DEGs) were analysed using the DESeq2 package (|fold change| ≥ 1.5, P < 0.05).

### Gene-set enrichment analysis and gene ontology analysis

GSEA was performed using the Broad Institute web platform by pre-ranking the RNA-seq list based on log2-fold change. Gene ontology analysis and KEGG pathways analysis were performed with Gene Ontology Resource (http://geneontology.org/) and R package “clusterProfiler” to assess the biological functions of this subset of DEGs [31].

### Cell proliferation assay and flow cytometry analysis

Cell proliferation was measured by CCK-8 (Dojindo Laboratories, Kumamoto, Japan) following the manufacturer’s instructions. The optical absorbance was measured at 450 nm by a plate reader (BioTek Instruments, Inc., Winooski, VT, USA).

Antibodies PE-conjugated CD11b were purchased from BD PharMingen (CA, USA). Total cells were Fcblocked and stained with indicated combinations of antibodies for 30 min on ice, then washed three times and resuspended in 1% FBS/PBS. For cell cycle assays, total cells were harvested and washed three times in PBS (pH 7.4), followed by fixation with precooled 70% ethanol overnight at 4 °C. Ethanol-fixed cells were centrifuged at 1000 rpm for 5 min, washed twice with PBS, and then incubated with 0.5 mL PBS containing 10 g/mL RNase A and 50 g/mL propidium iodide (PI, Sigma-Aldrich, St. Louis, MO, USA) for 30 min in the dark at 4 °C. The flow cytometric data were collected on a BD LSRFortessa flow cytometer (BD Biosciences, San Jose, CA, USA) and analyzed using FlowJo software or ModFit LT3.0 software.

### Statistical data

Data are presented as mean ± SD. A 2-tailed Student t-test was performed for all compared data by using GraphPad Prism version 7. A P < 0.05 was considered to be statistically significant (*), a P < 0.01 was regarded as statistically very significant (**).

## Results

### KDM3B is highly expressed in hematopoietic malignance and correlated with the favorable prognosis of AML patient

**Fig. 1:**
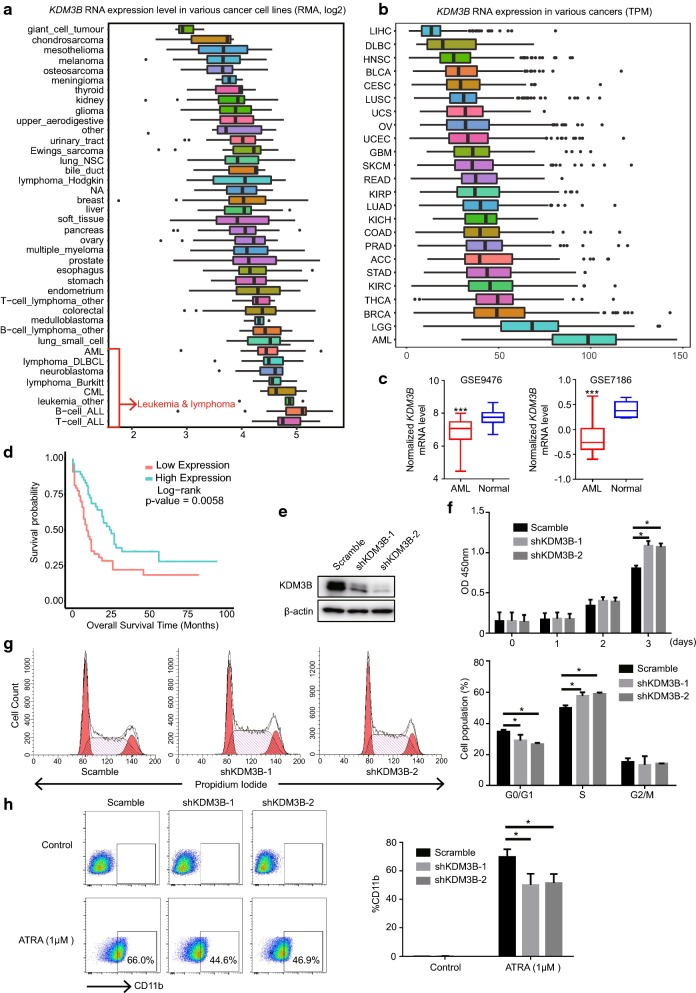
KDM3B is required for the growth and granulocytic differentiation of NB4 cells. **a**, **b** The mRNA expression levels of KMD3B across various types of human cancers. Data were retrieved from CCLE dataset (**a**), which contains RNA-seq of 1457 human cancer cell lines, and the TCGA dataset (**b**). Arrow, leukemia and lymphoma cells. **c** KDM3B mRNA levels in AML patients and normal counterparts were compared (raw data retrieved from from GSE9476 and GSE7186. **d** Overall survival of AML patient cohort with regard to KDM3B mRNA levels. **e** Effects of stable KDM3B-knockdown in APL NB4 cells by Western blot analysis. **f** Knock-down of KDM3B promotes NB4 cell proliferation. CCK8 assay was performed to determine the cell growth of NB4 cells transfected with scramble control or shKDM3B. **g** Cell cycle was determined by flow cytometry analysis. The bar chart represented the percentage of cells in G0/G1, S, or G2/M phases respectively. **h** Flow cytometric analysis of CD11b cell-surface expression in NB4 cells transfected with scramble control or shKDM3B followed by treatment with or without ATRA. Values are derived from three independent experiments, and data were mean ± SD. *P < 0.05, **P < 0.01, ***P < 0.001

First, the expression of KDM3B across tumor types were tested. By analyzing the expression of the Broad Institute’s CCLE cell lines, we found that KDM3B was significantly highly expressed in human leukemia and lymphoma cell lines compared to other tumor cell lines (Fig. 1a). This observation was further confirmed in tumor patients from The Cancer Genome Atlas (TCGA) dataset. Compared to other tumor type’s patients, AML patients had the highest expression of KDM3B (Fig. 1b). Those results emphasized the significance of KDM3B in hematopoietic malignance.

Then, we determined the expression of KDM3B in normal and malignant hematopoietic cells. The data of KDM3B expression in two cohorts of AML patients were downloaded from GEO datasets with GEO number GSE9476 and GSE7186. An abnormal KDM3B expression were observed in AML cells. Compared to normal cells, KDM3B mRNA level were significantly reduced in AML blasts cells (Fig. 1c). Importantly, using the TCGA data, we found that low expression of KDM3B was an unfavorable prognosis in AML patients (P = 0.0058) (Fig. 1d). These results were consistent with the observation of loss of KDM3B chromosome region in AML and highlighted the inhibitory roles of KDM3B in hematopoietic malignancies.

### Silencing KDM3B promotes tumor cells growth and inhibits ATRA induced NB4 cell differentiation

To further explore the functions of KDM3B in hematopoietic malignancies, we knocked down KDM3B expression in NB4 cells. NB4 cells were derived from granulocytic M3/APL subtype of AML, which expressed PML/RARα fusion protein. Lentivirus-mediated short hairpin RNAs (shKDM3B-1 and shKDM3B-2) targeting KDM3B or a negative control (scramble) were transfected into NB4 cells. Western blot were used to test the knock down efficiency. Compared to the scramble control, KDM3B was significantly down regulated in NB4 cell transfected with shKDM3B-1 and shKDM3B-2 (Fig. 1e).

The cell proliferation was examined in KDM3B-knockdown NB4 cells. Results showed that shRNA knock-down of endogenous KDM3B did significantly promoted the proliferation of NB4 cells compared with the control (Fig, 1f). Cell cycle distribution was determined using cytometry analysis. NB4 cells with shKDM3B had an obvious increased percentage of S phase cells (Fig. 1g), suggesting the high DNA synthesis and cell proliferation rate when KDM3B was deficient.

In addition, we investigated the potential roles of KDM3B in ATRA mediated leukemia cell differentiation by flow cytometry analysis using CD11b, a granulocytic differentiation marker. Notably, KDM3B-knockdown NB4 cells clearly had a low expression level of the differentiation marker CD11b despite ATRA treatment (Fig. 1h). Collectively, these results indicated that KDM3B was an important contributor to APL cell survival and differentiation.

### Silencing KDM3B globally increases the level of H3K9me1 and reduces the level of H3K9me2

**Fig. 2:**
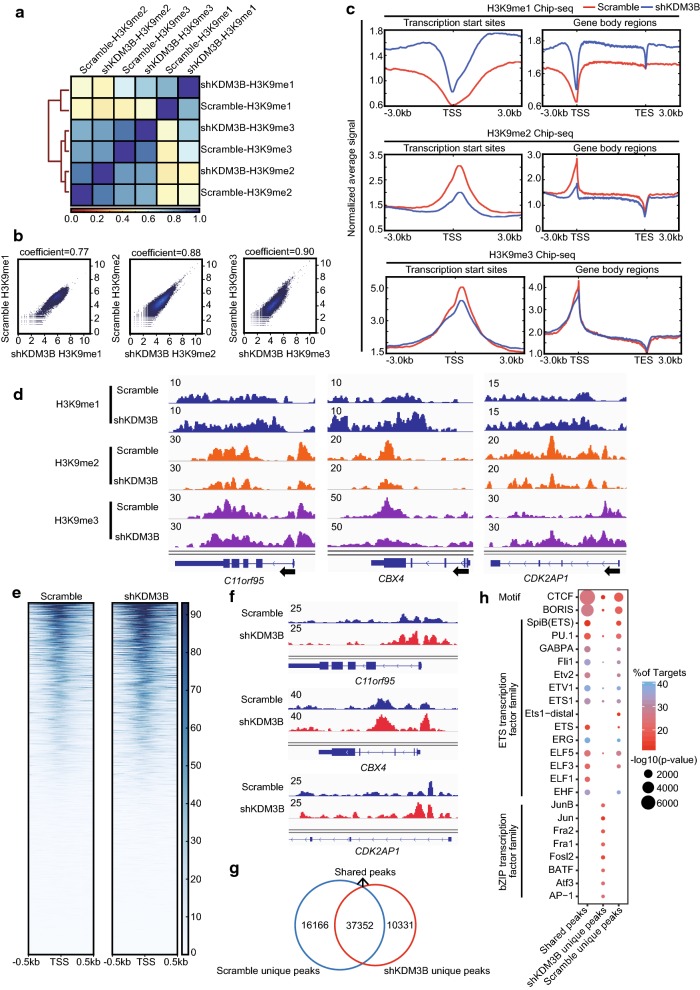
KDM3B is essential to regulate H3K9me1/me2 histone modifications and chromatin accessibility. **a** Correlation heatmap of genome-wide enrichment for H3K9me1, H3K9me2 or H3K9me3 ChIP-seq data in NB4 cells expressing scrambled control versus shKDM3B, organized and ordered using hierarchical clustering, is shown. Each modification type was then scaled to 0–1 and the R coefficient was determined with Spearman’s correlation. Colors indicate no correlation (red), intermediate correlation (yellow), and strong correlation (blue). **b** Plots showing the genome-wide correlation from H3K9me1, H3K9me2 or H3K9me3 ChIP-seq in NB4 cells expressing scrambled control versus shKDM3B, respectively. **c** Signal intensity plot representing changes in H3K9me1, H3K9me2 and H3K9me3 ChIP-seq signal at promoters regions or gene body following silencing of KDM3B in NB4 cells. The enriched regions were extended ± 3 kb from their midpoint. **d** Genome browser tracks of H3K9me1, H3K9me2 and H3K9me3 ChIP-seq data at the *C11orf95*, *CBX4* and *CDK2AP1* loci following silencing of KDM3B in NB4 cells. **e** Chromatin accessibility in NB4 cells expressing scrambled control versus shKDM3B at promoters regions. The enriched regions were extended ± 0.5 kb from their midpoint. **f** Genome browser tracks of ATAC-seq data at the *C11orf95*, *CBX4* and *CDK2AP1* loci following silencing of KDM3B in NB4 cells. **g** Venn diagram representing overlap of accessible sites in NB4 cells expressing scrambled control versus shKDM3B. **h** TF motif analysis of different regions in NB4 cells expressing scrambled control versus shKDM3B in all chromatin accessible sites. The scale of the circles represents motif enrichment

KDM3B, which belongs to the subfamily of proteins containing a JmjC domain, has been previously shown to participate in H3K9me1/me2 demethylation activities at specific regions within the genome. Although several studies have tried to assess KDM3B activity, the conclusions have been contradictory [22, 24, 32]. Therefore, the H3K9 methylation regulated by KDM3B in genome-wide were investigated by ChIP-seq in NB4 cells that stably expressed either the scramble control or shKDM3B. Correlation clustering analyses showed that H3K9me1/me2/me3 binding in NB4 cells with shKDM3B or scramble control was highly correlated (Spearman’s correlation coefficient = 0.77, 0.88 and 0.90, respectively) (Fig. 2a, b). From the metagene plots, we observed that KDM3B silencing increased the levels of H3K9me1 and reduced the levels of H3K9me2 in both the TSS region and the gene-body region, while global levels of H3K9me3 remained largely variable (Fig. 2c). For example, we observed substantial changes in H3K9me1/me2/me3 occupancy at the *C11orf95*, *CBX4* and *CDK2AP1* loci (Fig. 2d). These findings were consistent with publications showing that KDM3B is a H3K9me1/me2 demethylase in human tissues.

To further investigate the genome-wide accessible regions regulated by KDM3B, ATAC-seq was used. As shown in the heatmap, KDM3B silencing dramatically increased the global chromatin accessibility in NB4 cells (Fig. 2e). Browser track representations showed increased chromatin accessibility of *C11orf95*, *CBX4* and *CDK2AP1* at three loci (Fig. 2f).

Next, transcription factors associated with KDM3B were identified. shKDM3B-specific open chromatin regions were selected for further study (Fig. 2g). By using motif analysis software HOMER, we found that NB4 cells with shKDM3B-specific open chromatin regions were enriched with binding sites for transcription factors known as members of bZIP transcription factor and ETS transcription factor families (Fig. 2h). bZIP transcription factors including AP-1, c-Jun, Jun-B, Atf3, BATF, Fra1 and Fra2, play important roles in positive regulation of transcription by RNA polymerase II. The ETS transcription factors family, especially the PU.1 factor, is a master regulator of myeloid differentiation. Interestingly, the CTCF factor was most enriched in ATAC-seq peaks (Fig. 2h). Previous results have suggested that transcription factor CTCF is associated with open chromatin regions. Taken together, our results showed that KDM3B specifically regulated chromatin accessibility and H3K9me1/me2 modification and taht these regulations are crucial for the NB4 leukemia cell survival and differentiation.

### The genes activated after KDM3B silencing are located in chromatin accessible regions

**Fig. 3:**
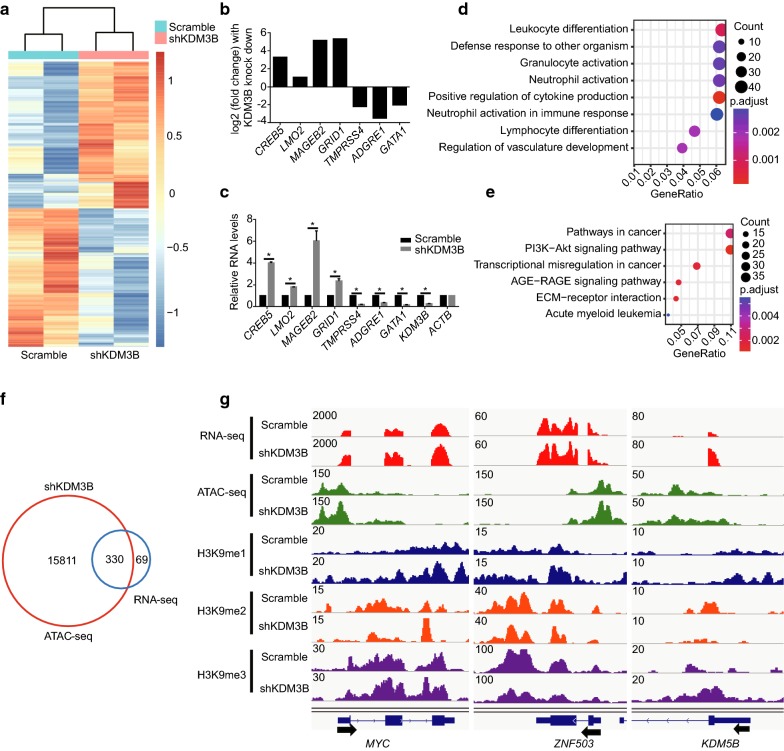
Integration of ATAC-seq data with RNA-seq for the activated genes after KDM3B silencing in NB4 cells. **a** Heatmap of differentially expressed genes (defined as |fold change| ≥ 1.5, with a P-value < 0.05) in KDM3B-knockdown NB4 cells relative to scrambled control cells. **b** Histogram showing a subset of the genes identified differentially expressed in NB4 cells expressing scrambled control versus shKDM3B from RNA-seq data. **c** RT-qPCR validation of differentially expressed genes in “B” following silencing of KDM3B in NB4 cells. Relative mRNA levels were calculated by 2^−ΔΔCT^ method and normalized to ACTB (β-actin). **d** GO analysis performed on the differentially expressed genes. **e** Pathway analysis using KEGG was performed on the genes differentially expressed. **f** Venn diagrams indicating the proportion of genes that differentially expressed in NB4 cells expressing scrambled control versus shKDM3B based on RNA-seq data that have ATAC-seq peaks (identified only in KDM3B knock-down NB4 cells). **g** Genome browser tracks of RNA-seq data; ATAC-seq data; H3K9me1, H3K9me2 and H3K9me3 ChIP-seq data at the *MYC*, *ZNF503* and *KDM5B* loci following silencing of KDM3B in NB4 cells. Values are derived from three independent experiments, and data were mean ± SD. *P < 0.05

We used RNA-seq to further study the regulatory mechanisms of KDM3B. A P-value < 0.05 and |fold change| ≥ 1.5 were set to be the standards to identify KDM3B regulatory genes to be compared against the scramble control. According to these parameters, 774 differentially expressed genes were acquired, of which 399 genes were upregulated and 357 genes were down regulated in the shKDM3B NB4 cells (Additional file 2). All the genes are depicted in heatmap (Fig. 3a). A panel of the top hits from our RNA-seq data was validated by conventional qPCR (Fig. 3b, c). We used Gene Ontology (GO) classification and KEGG to explore the biological functions of the KDM3B regulatory genes. The GO classification included leukocyte differentiation and neutrophil activation (Fig. 3d). The KEGG pathway analysis showed significant enrichment of the pathways typically found in cancer (Fig. 3e).

Furthermore, to determine whether chromatin open regions from the ATAC-seq analysis correlated with the KDM3B target gene expression, we integrated our ATAC-seq data with and RNA-seq data. Venn diagrams showed that 83% of upregulated genes in KDM3B-knockdown NB4 cells had open chromatin regions (Fig. 3f). These results suggest that open chromatin may be a predictor of gene activation in NB4 cells. In contrast, only 2% of ATAC-seq peaks were mapped to differentially expressed genes (Fig. 3f). As shown by the genomic browser tracks (Fig. 3g), chromatin openness and MYC expression were markedly increased, as shown in the left panel. However, an increased ATAC-seq signal did not significantly alter ZNF503 transcript levels (middle panel), and increased KDM5B expression did not accompany by increased chromatin accessibility (right panel). These results concur with the growing understanding that gene activation depends on multiple regulatory regions, many of which are located far from the gene locus itself.

### KDM3B facilitates the transcriptional alterations induced by ATRA

**Fig. 4:**
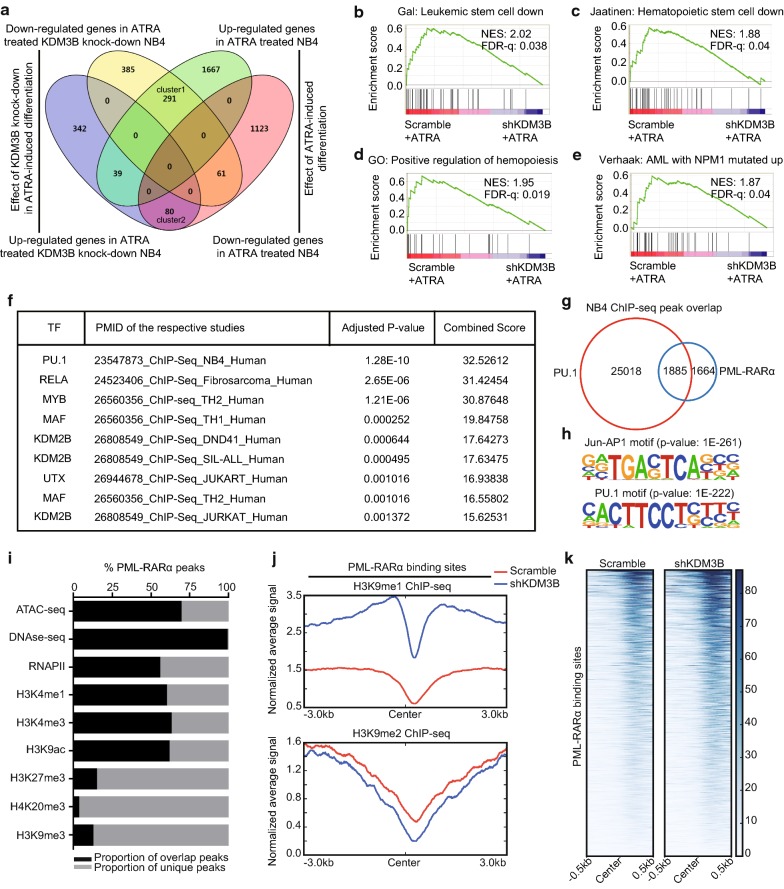
KDM3B influences the expression of ATRA-regulated genes in differentiation of NB4 cell. **a** Venn diagram illustrates the number of genes regulated by ATRA, the genes influenced by KDM3B in ATRA-induced differentiating, and the overlaps of these 2 gene sets. 291 genes are less induced by ATRA in KDM3B-knockdown NB4 cells than normally expected (cluster1), 80 genes are more up-regulated in KDM3B-knockdown NB4 cells as compared with the control (cluster 2). **b**–**e** GSEA of the expressing profile of NB4 cells transfected with scramble control or shKDM3B followed by treatment with ATRA using a Leukemic stem cell down-regulated signature (**b**), a Hematopoietic stem cell down-regulated signature (**c**), a positive regulation of hemopoiesis-associated signature (**d**), and a AML with NPM1 mutated-associated signature (**e**). **f** ChIP enrichment analysis of differentially expressed genes in KDM3B-knockdown NB4 cells relative to scrambled control cells followed by treatment with ATRA. The top 9 ranked transcription factors are shown (ranked by adjusted P-value). **g** Overlap of PU.1 and PML/RARα binding revealed by ChIP-seq. The peak numbers of each category are shown on the venn diagram. **h** De novo motif analysis of PML/RARα binding sites. Significantly enriched motifs are shown. **i** PML/RARα binding sites overlap with the active and repress histone modification. **j** Signal intensity plot representing changes in H3K9me1 and H3K9me2 ChIP-seq signal at PML/RARα binding regions followed by silencing of KDM3B in NB4 cells. The enriched regions were extended ± 3 kb from their midpoint. **k** Chromatin accessibility in NB4 cells expressing scrambled control versus shKDM3B at PML/RARα binding regions. The enriched regions were extended ± 0.5 kb from their midpoint

ATRA is widely used as a differentiating agent in the clinic [33, 34]. Notably, KDM3B-knockdown NB4 cells clearly inhibited ATRA-mediated APL differentiation (Fig. 1h). To further characterize the relationship between the KDM3B modulatory effect and gene expression changes related to the APL differentiation process, the differential gene expression patterns of KDM3B-knockdown NB4 cells were compared against those of the scrambled control cells in the presence of ATRA, and the differentiated control NB4 cells were compared with the undifferentiated NB4 cells, as schematically depicted in Fig. 4a (Additional files 3, 4). As shown by the venn diagram, 291 ATRA-induced genes showed reduced expression in KDM3B-knockdwon NB4 cells (cluster 1), while only 80 ATRA-inhibited genes were upregulation in cells lacking KDM3B (cluster2). Thus, KDM3B has an important facilitating role in the induction of certain genes characteristic of the differentiation process.

Moreover, we performed gene set enrichment analysis (GSEA) to define the functions of the 1198 differential expression genes of the KDM3B-knockdown NB4 cells versus those of the scrambled control cells induced by ATRA (P-value < 0.05 and |fold change| ≥ 1.5). In line with the flow cytometric results, knocking down KDM3B led to suppressed expression of a leukemia stem cell (LSC) gene signature and a hematopoietic stem cell gene signature (Fig. 4b–d). We also found a negative association between the NPM1-mutated AML gene signature and KDM3B-knockdown gene signature in NB4 cells (Fig. 4e).

To further characterize the putative transcription factors that control the aberrant expression of these genes, we performed an enrichment analysis of the ChIP-X (ChEA) database with Enrichr. Among the transcription factors in the database, PU.1 was ranked as one of the most potent transcription factors involved in regulating the differentially expressed genes (Fig. 4f).

We previously demonstrated that PML/RARα is recruited to PU.1-bound chromatin containing RARE half and PU.1 sites [25, 26]. In the present study, we found that over 53% (1885/3549) of the PML/RARα binding regions are targeted by PU.1 in NB4 cells (Fig. 4g). De novo motif analysis revealed that the PU.1 and Jun-AP1 binding motifs are the most significant motifs found in PML/RARα bound regions (Fig. 4h). It has been suggested that APL-specific PML/RARα fusion protein is bound to chromatin open regions [35]. Integrating high-throughput sequencing data from a variety of ChIP-seq experiments and results from analysis of the accessible chromatin landscapes, including those based on DNase-seq and ATAC-seq, we found that the vast majority of PML/RARα binding sites overlap with epigenetic marks of active chromatin (H3K4me3, H3K4me1, H3K9ac and RNAPII), while a minority overlap with inhibitory chromatin marks (H3K27me3, H4K20me3 and H3K9me3) (Fig. 4i). Combined with the results previously obtained (Fig. 2), we speculate that KDM3B might contribute to genome-wide binding of PML/RARα. Notably, when KDM3B was silenced, the PML/RARα binding sites were occupied by a substantially increased number of H3K9me1 marks, and a modest loss of H3K9me2 signal was observed (Fig. 4j). In addition, KDM3B silencing in NB4 cells led to increased chromatin accessibility in PML/RARα binding regions (Fig. 4k). Together, these data strongly implied that the KDM3B expression is closely linked to the state of normal granulocytic differentiation by altering chromatin accessibility.

### KDM3B regulates ATRA-induced degradation of PML/RARα and chromatin state in NB4 cells

**Fig. 5:**
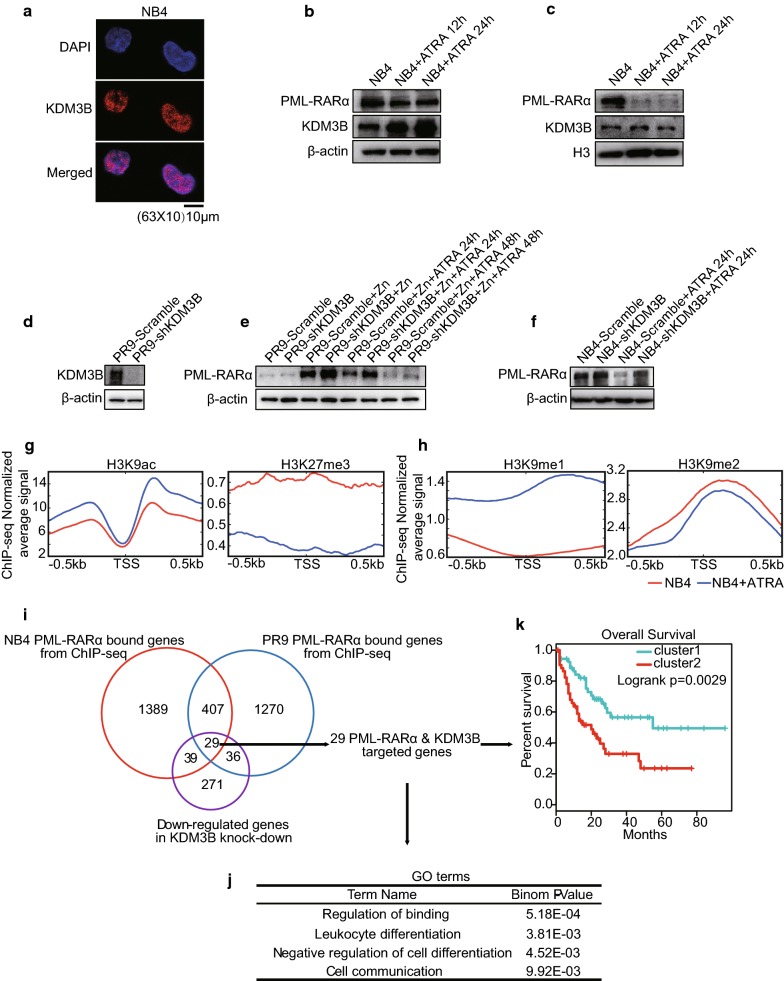
KDM3B is associated with PML/RARα oncoprotein during NB4 cell differentiation. **a** Confocal staining of KDM3B in NB4 cells. Nuclei were counterstained with DAPI. Representative images are shown. Scale bar: 10 μm. **b**, **c** Western blot analyses of KDM3B and PML/RARα in NB4 cells at total/nuclear protein level, followed by treatment with ATRA. β-actin was used as a loading control for total protein (**b**), histone H3 was used for nuclear proteins (**c**). **d** Effects of stable KDM3B-knockdown in PR9 cells by Western blot analysis. **e** Western blot analyses of PML/RARα in PR9 cells transfected with scramble control or shKDM3B, with or without Zn^2+^ pretreatment for 4 h, followed (or not) by treatment with ATRA. **f** Western blot analyses of PML/RARα in NB4 cells transfected with scramble control or shKDM3B followed (or not) by treatment with ATRA. **g**, **h** Signal intensity plot representing changes in H3K9ac, H3K27me3, H3K9me1 and H3K9me2 ChIP-seq signal at promoters regions in NB4 cells followed by treatment with ATRA. The enriched regions were extended ± 0.5 kb from their midpoint. **i** Strategy and workflow of integrative analysis to identify 29 target genes directly regulated by KDM3B and PML/RARα. **j** GO analysis performed on KDM3B and PML/RARα co-regulated 29-gene signature. **k** Overall survival of AML patient cohort with regard to the concomitant KDM3B and PML/RARα co-regulated 29-gene signature. Values are derived from three independent experiments, and data were mean ± SD. *P < 0.05

In our study, we confirmed that KDM3B is required for ATRA-induced granulocytic differentiation in NB4 cells. Then, we sought to determine whether KDM3B is involved in the regulation of expression of APL-specific PML/RARα fusion protein expression. To test this possibility, we first used immunostaining to detemine that KDM3B is primarily distributed in the nucleus of NB4 cells (Fig. 5a). Second, we examined KDM3B expression in an ATRA-induced differentiation time series experiment using NB4 cells. Upregulation of KDM3B is clearly observed at total protein level, whereas the downregulation of KDM3B is observed at nuclear protein level (Fig. 5b, c). Finally, we investigated whether the inhibition of KDM3B can attenuate the ATRA-induced clearance of PML/RARα. It was shown that knocking down KDM3B impaired the degradation of PML/RARα during cell differentiation in both PR9 cells and NB4 cells (Fig. 5d–f).

Few studies have verified that ATRA treatment leads to acetylation of histones H3 and H4, which induces widespread chromatin opening with low modification levels of epigenetic marks of suppressed chromatin [35, 36], such as H3K27me3 (Fig. 5g). In addition, we also found that ATRA induces an increase in H3K9me1 and a decrase in H3K9me2 near the TSS regions in the NB4 cells (Fig. 5h), a finding that is consistent with epigenetic changes induced by knocking down KDM3B. Together, these data indicate that KDM3B is not only an ATRA-responsive gene but is also a contributor to sustained PML/RARα levels and chromatin state maintenance during cell differentiation, showing a close correlation between KDM3B levels and PML/RARα regulation.

To improve the sensitivity and specificity of potential target gene identification to find those that are regulated by KDM3B and PML/RARα, we conducted an integrated analysis of the ChIP-seq DNA–protein binding and RNA-seq differential expression data (Fig. 5i). PML/RARα ChIP-seq data were derived from the public data on NB4 cells and PR9 cells and analyzed. Altogether, we identified a set of 436 PML/RARα-binding genes shared by these two cell types. Among the PML/RARα binding targets that we identified, 29 genes showed a significant decrease in mRNA when KDM3B was knocked down (Additional file 5). These 29 genes were considered as the PML/RARα-KDM3B coregulated gene signature, which included key regulators of cell fusion and substrate binding (Fig. 5j) and contributed to prediction of the overall survival for patients with AML (Fig. 5k).

## Discussion

**Fig. 6:**
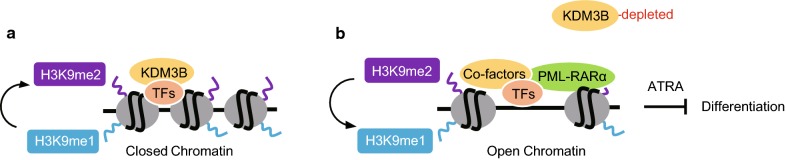
A proposed model for KDM3B and PML/RARα in APL cell differentiation with KDM3B existence (**a**) or depletion (**b**), respectively. See “Discussion” for details

In this study, we provide several lines of evidence that KDM3B, H3K9me1/me2 demethylase, exerted a key role in the epigenetic regulation and cellular responses to ATRA treatment in APL by using a genetically defined human APL model of PML/RARα positive NB4 cells. We propose a mechanism that KDM3B was required to maintain a compact chromatin state in proper development and function of APL (Fig. 6a). The absence of KDM3B could alter H3K9me1/me2 modification level companied with increasing of chromatin accessibility and inhibit ATRA-induced degradation of PML/RARα and, consequently, allow “easier” chromatin access and binding of PML/RARα protein in APL, which eventually leads to attenuate neutrophil differentiation of APL cells (Fig. 6b).

Previous studies have demonstrated that KDM3B is located at a locus (5q31 chromosome region) commonly deleted in approximately 15% of primary MDS cases, 10% of AML cases, and 40% of the therapy-induced cases of MDS/AML [19, 20, 37]. In addition, KDM3B−/− mice displayed abnormal phenotypes in the hematopoietic system [32]. Therefore, aberrantly low expression of KDM3B is thought to have latent functions in AML and MDS. Unexpectedly, it was recently shown that KDM3B acts as an H3K9me1/me2 demethylase and induces leukemic transformation in AML subtypes, such as HL-60 (AML FAB M2) and MUTZ-8 (AML-derived) [22, 24]. However, there is controversy surrounding the mode by which the marks are distributed on the histones, as some authors suggested that there were no remarkable changes in H3K9me1 after KDM3B was knocked down, while some others report finding a negative correlation between H3K9me1 and KDM3B. Considerating that all these studies were focused on specific chromatin loci, the underlying mechanism of KDM3B combined with the genomic landscape of H3K9me1/me2 modifications and chromatin remodeling, deserves further exploration.

The levels of H3K9me2/me3 have been found to be enriched in nongenic regions, which resulted in heterochromatin formation and epigenetic silencing, while H3K9me1 levels were enriched at active genes and open chromatin [38, 39]. Moreover, histone demethylation and chromatin decondensation can lead to dysregulated gene expression and transcriptional activation of gene targets [8, 27, 40]. Our results suggest that knocking down KDM3B could increase the level of H3K9me1 and reduce the level of H3K9me2 in both the TSS region and gene-body region. This finding supports the notion that KDM3B induces the specific demethylation of H3K9me1 and H3K9me2. In addition, knocking down KDM3B globally enhanced chromatin accessibility, which facilitated transcription release. Furthermore, by integrating ATAC-seq data with RNA-seq data using KDM3B-knockdown NB4 cells, our results could also reflect the fact that opening chromatin only in certain regions is not sufficient for gene activation.

In the case of KDM3B-knockdown cells, we found that the CTCF consensus motif was significantly enriched in open chromatin regions. Importantly, CTCF is a widely expressed transcription factor and a “gatekeeper factor” that regulates transition from dormancy to activation in hematopoietic stem cell (HSC) development and controls stem cell pool throughout life [41–46]. Moreover, after KDM3B silencing, selective open chromatin regions were enriched in bZIP transcription factor, which plays an important roles in AML development [47–49]. Thus, further study of these transcription factors in APL is expected to reveal insights into the temporally and spatially regulated chromatin structure that programs in myeloid differentiation.

PML/RARα is the hallmark fusion oncoprotein implicated in APL pathogenesis and results in the attenuation of gene expression needed for myeloid differentiation [34]. Previous studies have shown that the majority of PML/RARα binding sites tend to be in regions of accessible chromatin with some hallmarks of active chromatin [25, 35, 36]. Moreover, ATRA exerts its therapeutic effect by promoting the degradation of the PML/RARα oncogenic protein, which subsequently promotes cell differentiation [33, 50]. In particular, the observations in this study indicated that ATRA-induced degradation of PML/RARα increases KDM3B expression at the total protein level while decreasing of KDM3B expression at the nuclear protein level. In addition, knocking down KDM3B impairs the degradation of PML/RARα during cell differentiation, suggesting the positive feedback between PML/RARα and KDM3B. All of these results, as discussed above, strongly suggest that KDM3B plays a critical role in regulating ATRA treatment in APL cells via KDM3B mediated chromatin opening.

In summary, our studies suggest that histone demethylase KDM3B correlates with APL disease and drug resistance, in addition, our data provide a novel insight into PML/RARα-driven APL pathogenesis and treatment. In this study, APL was used as an AML model to investigate the functions of KDM3B. These results may be achieved in other AML types. We show that, compared with those in normal cells, KDM3B mRNA levels were significantly reduced in AML blast cells (Fig. 1c), suggesting that KDM3B is critical for overall AML cell survival. Future studies using multiomics resource are warranted to investigate the role of KDM3B in other types of leukemia from.

## Conclusion

Histone demethylase KDM3B is considered to play a critical role in leukemogenesis. To the best of our knowledge, this study is the first attempt to probe the detailed genetic and epigenetic mechanisms underlying the regulation of KDM3B in the development of APL from the perspectives of multi-layer omics. Results of the present study demonstrate that KDM3B exerts anti-APL effect by directly modulating H3K9me1/me2 levels to maintain compact chromatin status, but also indicate the interaction between KDM3B and PML/RARα regulates degradation of PML/RARα. These findings will provide insight into PML/RARα-driven APL pathogenesis, and theoretical and experimental data for the treatment regimens and target therapy of APL.

## Supplementary information

**Additional file 1.** Quantitative PCR primers and details of antibodies.

**Additional file 2.** Differentially expressed genes between KDM3B-knockdown NB4 versus scrambled control NB4 cells.

**Additional file 3.** Differentially expressed genes between KDM3B-knockdown NB4 versus scrambled control NB4 cells followed with ATRA treatment.

**Additional file 4.** Differentially expressed genes between ATRA-induced differentiated control NB4 versus undifferentiated NB4 cells.

**Additional file 5.** PML/RARα-KDM3B coregulated 29-genes signature.

## Data Availability

H3K9ac, H3K27me3, PML and RARα ChIP-seq data for NB4 and PR9 cells were obtained from GSE18886 [36]. Microarray gene expression data for AML patients were obtained from GSE9476 and GSE7186 [51, 52]. The data for ChIP-seq and RNA-seq analyses can be found at Gene Expression Omnibus GSE137105.

## Abbreviations

KDM3B: histone lysine-specific demethylase 3B
APL: acute promyelocytic leukemia
ATRA: all-trans retinoic acid
PML: promyelocytic leukemia protein
MDS: myelodysplastic syndromes
AML: acute myeloid leukemia
TSS: transcription start site
TCGA: The Cancer Genome Atlas
GSEA: gene set enrichment analysis
ChIP: chromatin immunoprecipitation
NGS: next-generation sequencing
